# Explaining evolution via constrained persistent perfect phylogeny

**DOI:** 10.1186/1471-2164-15-S6-S10

**Published:** 2014-10-17

**Authors:** Paola Bonizzoni, Anna Paola Carrieri, Gianluca Della Vedova, Gabriella Trucco

**Affiliations:** 1 Dipartimento di Informatica Sistemistica e Comunicazione, Università degli Studi di Milano-Bicocca, viale Sarca 336, Milano, Italy; 2 Dipartimento di Informatica, Università degli Studi di Milano, Via Bramante 65, Crema, Italy

**Keywords:** perfect phylogeny, persistent perfect phylogeny, fixed-parameter complexity

## Abstract

**Background:**

The perfect phylogeny is an often used model in phylogenetics since it provides an efficient basic procedure for representing the evolution of genomic binary characters in several frameworks, such as for example in haplotype inference. The model, which is conceptually the simplest, is based on the infinite sites assumption, that is no character can mutate more than once in the whole tree. A main open problem regarding the model is finding generalizations that retain the computational tractability of the original model but are more flexible in modeling biological data when the infinite site assumption is violated because of e.g. back mutations. A special case of back mutations that has been considered in the study of the evolution of protein domains (where a domain is acquired and then lost) is persistency, that is the fact that a character is allowed to return back to the ancestral state. In this model characters can be gained and lost at most once. In this paper we consider the computational problem of explaining binary data by the Persistent Perfect Phylogeny model (referred as PPP) and for this purpose we investigate the problem of reconstructing an evolution where some constraints are imposed on the paths of the tree.

**Results:**

We define a natural generalization of the PPP problem obtained by requiring that for some pairs (character, species), neither the species nor any of its ancestors can have the character. In other words, some characters cannot be persistent for some species. This new problem is called Constrained PPP (CPPP). Based on a graph formulation of the CPPP problem, we are able to provide a polynomial time solution for the CPPP problem for matrices whose conflict graph has no edges. Using this result, we develop a parameterized algorithm for solving the CPPP problem where the parameter is the number of characters.

**Conclusions:**

A preliminary experimental analysis shows that the constrained persistent perfect phylogeny model allows to explain efficiently data that do not conform with the classical perfect phylogeny model.

## Background

Character-based phylogeny is a broad notion to represent an evolutionary history describing the ancestral relationships among extant taxa or individuals. Recent applications show that the model can be applied to study the evolution of mutations related to various genomic information, such as protein domains [1] or markers in tumors. Thus in our formulation, it is not important whether we are actually studying taxa or individuals or other genomic data. We will follow the usual convention of calling *species *the units of study. The main element of this notion is that the instance is also made of a set of *characters*, and each species is in a specific state for each character [2]. The goal is to find a phylogeny where the known species are the leaves, and the internal nodes are labeled--just as the leaves--by a state for each character. For each edge (*x, y*) of the phylogeny, the mutated characters along the edge are those whose states are different in *x *and *y*. The simplest case is when all characters are binary, that is only two states (0 and 1) are possible, modeling the situation when each species has or does not have a given feature, such as wings (a phenotypical trait) or the mutation encoding lactase persistence (a genotypical trait).

Moreover, we are assuming a coalescent model, that is the fact that a characteristic shared by a set of species can be traced back to a single ancestral species. Assuming that the state 1 encodes the fact that a species has a given character (for example, the fact that the species has acquired a given mutation), the coalescent model implies that the phylogeny is directed. Restrictions on the type of changes from zero to one and vice versa lead to a variety of specific models [3].

The perfect phylogeny is one of the most investigated coalescent models [2]. Conceptually the model is based on the infinite sites assumption, that is no character can mutate more than once in the whole tree. The binary perfect phylogeny problem has received much attention, culminating with the linear time algorithm when all data is known [4] and an efficient algorithm when the input data is incomplete [5]. While the infinite sites assumption is quite restrictive, the perfect phylogeny model turned out to be splendidly coherent within the haplotyping problem [6,7], where we want to distinguish the two haplotypes present in each individual when only genotype data is given. More precisely, the interest here is in computing a set of haplotypes and a perfect phylogeny such that the haplotypes (i) label the vertices of the perfect phylogeny and (ii) explain the input set of genotypes. This context has been deeply studied in the last decade, giving rise to a number of algorithms [8,9]. Still, the perfect phylogeny model and the assumptions that have been central in the previous decades cannot be employed without adaptations or improvements. A first generalization in the literature allows for more states (but keeping the infinite sites assumption). In the general case, the problem is NP-hard [10], but it has an algorithm parameterized by the number of states [11,12]. The special cases when there are three or four possible states have more efficient algorithms [13-15].

Even allowing more states cannot explain the biological complexity of real data, when homoplasy events (such as recurrent mutations or back mutations) are present. Two cases where those limitations are evident are the study of carcinogenesis and protein domains. Carcinogenesis consists of the factors and mechanisms that cause the onset of cancer; it results from many combinations of mutations, but only a few, called progression pathways, seem to account for most human tumors [16]. The observation that tumors are evolving cell populations leads to phylogeny-based studies. At the same time the intrinsic nature of quickly and degenerately proliferating cancer cells, results in a relative high amount of sites with multiple mutations (i.e., in violations of the infinite sites assumption). A protein domain is a part of protein sequence and structure that can evolve independently of the rest of the protein chain. Many proteins consist of several structural domains, while a domain may appear in a variety of different proteins. In this case it is quite frequent to acquire a domain and then to lose it [17].

Thus a central goal of this paper is to find a model that is more widely applicable than the perfect phylogeny, while retaining its computational efficiency (in fact, more general models such as the Dollo and the Camin-Sokal models are NP-hard [3]). The problem of constructing phylogenies where the deviations from perfect phylogeny are small has been tackled under the name of near perfect phylogeny [11] or near perfect phylogeny haplotyping problems [18]. Especially the impossibility of losing a character that has been previously acquired is too restrictive, resulting in more elaborated models, such as the persistent character [1] and the General Character Compatibility [19,20].

More precisely, the Persistent Perfect Phylogeny model [21] allows each character to be lost (i.e., going from state 1 to 0) in at most an edge of the phylogeny, while the General Character Compatibility imposes some restrictions on the possible mutations (that is on the possible states labeling the endpoints of an edge), while allowing the input data to be a set of possible states for each character of a species. In this paper we combine the Persistent Perfect Phylogeny (PPP) and the General Character Compatibility (GCC), introducing the Constrained Persistent Perfect Phylogeny problem (CPPP) which generalizes the PPP by adding a constraint for some characters *c *in the input data, given by the fact they cannot be persistent for some species *s *(i.e., the state of *c *does not go from 1 to 0 for any edge lying on the path from the root to *s*). Since the CPPP problem is equivalent to a case of GCC whose complexity is still open [19,22], our results also apply to GCC.

Finally, we explore some algorithmic solutions for the CPPP problem. In particular, we give a polynomial time solution of the CPPP problem over matrices whose conflict graph has no edge. This result partially answer the open problem stated in [21] of determining the computational complexity of the PPP problem. In the paper we have run a preliminary experimental analysis showing that our method can manage successfully binary characters data incorporating back mutations. The results show that the algorithm performs efficiently on simulated matrices as well as on real data taken from the HapMap project.

### The persistent perfect phylogeny

Our approach follows [21] to which we refer the reader for a detailed discussion of PPP, while we give here only a cursory treatment. The input of the PPP problem is an *n *× *m *binary matrix *M *whose columns are associated with the set *C *= {*c*_1_, . . . , *c_m_*} of characters and whose rows are associated with the set *S *= {*s*_1_, . . . , *s_n_*} of species. Then *M*[*i, j*] = 1 if and only if the species *s_i _*has character *c_j_*, otherwise *M*[*i, j*] = 0. The character *c *is *gained *in the only edge where its state goes from 0 to 1 or, more formally, in the edge (*x, y*) such that *y *is a child of *x *and *c *has state 0 in *x *and state 1 in *y*. In this case the edge (*x, y*) is labeled by *c*_+_. Conversely, *c *is *lost *in the edge (*x, y*) if *y *is a child of *x *and the *c *has state 1 in *x *and state 0 in *y*. In the latter case the edge (*x, y*) is labeled by *c^−^*. For each character *c*, we allow at most one edge labeled by *c^− ^*[21,23].

**Definition 1 **(Persistent Perfect Phylogeny) Let *M *be an *n *× *m *binary matrix. Then a *persistent perfect phylogeny*, in short *p-pp*, for *M *is a rooted tree *T *such that:

1 each node *x *of *T *is labeled by a vector *l_x _*of length *m*;

2 the root of *T *is labeled by a vector of all zeroes, while for each node *x *of *T *the value *l_x_*[*j*] ∈ {0, 1} represents the state of character *c_j _*in tree *T*;

3 each edge *e *= (*v, w*) is labeled by at least a character;

4 for each character *c_j _*there are at most two edges *e *= (*x, y*) and *e*' = (*u, v*) such that *l_x_*[*j*] ≠ *l_y_*[*j*] and *l_u_*[*j*] ≠ *l_v_*[*j*] (representing a change in the state of *c_j_*). In that case *e, e*' occur along the same path from the root of *T *to a leaf of *T*; if *e *is closer to the root than *e*', then *l_x_*[*j*] = *l_v_*[*j*] = 0, *l_y_*[*j*] = *l_u_*[*j*] = 1, and the edge *e *is labeled $c_{j}^{+}$, while *e*' is labeled $c_{j}^{-}$;

5 each row *r *of *M *labels exactly one node *x *of *T*. Moreover the vector *l_x _*is equal to the row *r*.

Let *s *be a species and let *c *be a character such that, in a persistent perfect phylogeny *T*, the path from the root of *T *to *s *traverses an edge labeled *c^−^*. Then *c *is called persistent for *s *in *T*.

The Persistent Perfect Phylogeny problem asks to find, if it exists, a persistent perfect phylogeny for a given binary matrix *M*. We can restate the PPP problem as a variant of the Incomplete Directed Perfect Phylogeny [5] by transforming the complete input matrix into an incomplete matrix, called extended matrix.

**Definition 2 **(Extended Matrix) Let *M *be an instance of the PPP problem. The *extended matrix *associated with *M *is an *n *× 2*m *matrix *M_e _*over alphabet {0, 1, ?} which is obtained by replacing each column *c *of *M *by a pair of columns (*c*^+^, *c^−^*), where ? means that the value of such cell is not given. Moreover for each row *s *of *M *if *M*[*s, c*] = 1, then *M_e_*[*s, c*^+^] = 1 and *M_e_*[*s, c^−^*] = 0, while if *M*[*s, c*] = 0, then *M_e_*[*s, c*^+^] =? and *M_e_*[*s, c^−^*] =?.

In this case the characters (*c*^+^, *c^−^*) are called conjugate. Informally, the assignment of the *conjugate *pair (?, ?) in a species row *s *for two conjugate characters (*c*^+^, *c^−^*) means that character *c *could be persistent in species *s*, i.e., it is first gained and then lost. On the contrary, the pair (1, 0) means that character *c *is only gained by the species *s*. A *completion *of a pair (?, ?) associated to a species *s *and characters (*c*^+^, *c^−^*) of *M_e _*consists of forcing *M_e_*[*c*^+^, *s*] = *M_e_*[*c^−^, s*] = 0 or *M_e_*[*c*^+^, *s*] = *M_e_*[*c^−^, s*] = 1, while a partial *completion M_e _*is a completion of some of its conjugate pairs. Notice that *M *admits a persistent phylogeny if and only if there exists a completion of *M_e _*admitting a directed perfect phylogeny [21].

A fundamental contribution of [21], building upon [5], is to frame the problem as a graph theory question. We briefly recall here the two graphs that are used in the description of the algorithm.

**Figure 1:**
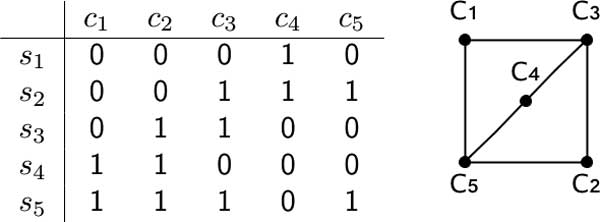
**A matrix and its conflict graph**.

Let *M *be a binary matrix and let *c*_1_, *c*_2 _be two characters of *M*. Then the configurations induced by the pair (*c*_1_, *c*_2_) in *M *is the set of ordered pairs (*M*[*s, c*_1_], *M*[*s, c*_2_]) over all species *S*. Two characters *c*_1 _and *c*_2 _of *M *are *conflicting *if and only if the configurations induced by such pair of columns is the set of all possible pairs (0, 1), (1, 1), (1, 0) and (0, 0). The *conflict graph G_c _*= (*C, E_c _*⊆ *C *× *C*) of a matrix *M *has vertices *C *and as edges the pairs (*c_i_, c_j_*) of conflicting characters (see Figure 1). We also need some graph-theoretic definitions. A graph without edges is called *edgeless*. A connected component is called *nontrivial *if it has more than one vertex.

The second graph used in the algorithm provides a representation of a completion of characters of an extended matrix. The *red-black graph G_RB _*= (*V, E*) associated to an extended matrix *M_e _*is the edge-colored graph where (i) the vertices are the species and the conjugate pairs of *M_e _*(that is for each two conjugate characters *c*^+ ^and *c^−^*, only *c *is a vertex of *G_RB_*), (ii) a pair (*s, c*) is a black edge iff the conjugate pairs *c*^+ ^and *c^− ^*are still incomplete in matrix *M_e _*and *M_e_*[*s, c*^+^] = 1 and *M_e_*[*s, c^−^*] = 0, (iii) (*s, c*) is a red edge iff the conjugate pairs *c*^+ ^and *c^− ^*are completed as *M_e_*[*s, c*^+^] = *M_e_*[*s, c^−^*] = 1.

An algorithm to compute a persistent perfect phylogeny

Let *T *be any persistent perfect phylogeny for a matrix *M *and consider a depth-first visit of *T*, the sequence of edge labels traversed during the visit is uniquely defined. The converse also holds, that is given a sequence *C *of edge labels, we can reconstruct the unique persistent perfect phylogeny *T *(if it exists) such that *C *is the sequence of edge labels traversed during a depth-first visit of *T *[21].

The main idea is that we associate a partial phylogeny *P *to each prefix of *C*, where each leaf *x *of *P *is labeled with the submatrix *M_x _*of *M_e _*such that *M_x _*has exactly the species and the characters that will be in the subtree of *T *rooted at *x*. Recall that each matrix *M_x _*has a graph representation given by the red-black graph. Then determining the next edge label to be added to the prefix of *C *is called to *realize *a character in the red-black graph representing *M_x _*as follows.

Let (*c*^+^, *c^−^*) be two conjugate characters of *M_e _*and let *G_RB _*its associated red- black graph. Let C(c) be the connected component of *G_RB _*containing the vertex *c*. A character is in one of three possible states: inactive (the initial state of all characters), active, and free. The *realization *of a character *c *in *G_RB _*consists of the following steps:

1 if *c *is inactive then:

(a) for each species s∉C(c), pose *M_e_*[*s, c*^+^] = *M_e_*[*s, c^−^*] = 0;

(b) for each species s∈C(c) if (*c, s*) is not an edge of *G_RB_*, add a red edge(*c, s*) and complete *M_e _*by posing *M_e_*[*s, c*^+^] = *M_e_*[*s, c^−^*] = 1;

(c) remove from *G_RB _*all black edges (*c, s*) and label *c active*.

2 else if *c *is active and *c *is connected by red edges to all species in C(c), then:

(a) all such red edges are deleted from *G_RB _*and *c *is labeled *free*;

Notice that when (i) *c *is free, or (ii) *c *is active but there exists a species s∈C(c) that is not connected to *c *by a red edge, none of the stated conditions hold. In these cases the realization is *impossible*.

**Figure 2:**
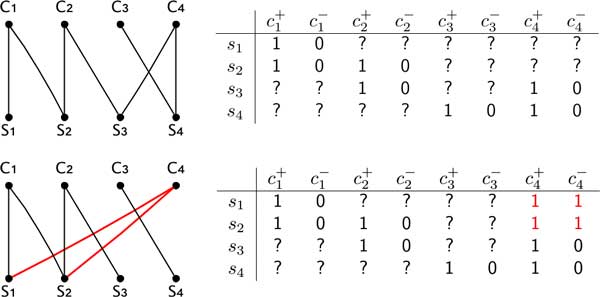
**The figure illustrates the realization of a character in a red-black graph associated to an extended matrix**. The canonical completion of the extended matrix after the graph operations is shown for the character *c*4.

**Figure 3:**
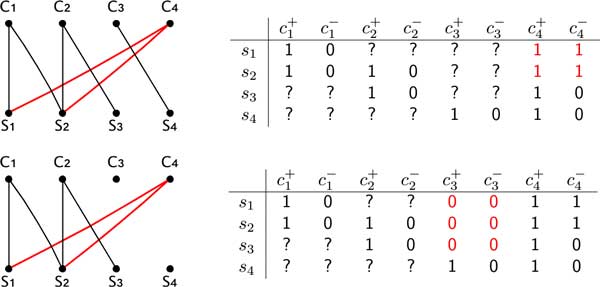
**The figure illustrates the realization of character *c*3 in the red-black graph associated to an extended matrix of the previous figure**. The canonical completion of the extended matrix is shown for the character *c*3.

Figures 2 and 3 illustrate the realization of characters. Moreover, isolated vertices of *G_RB _*correspond to leaves of the partial phylogeny *P *whose associated matrix has only one species; that instance is trivially solvable, therefore isolated vertices can be removed from *G_RB_*.

We recall that, to obtain an algorithm for PPP, it suffices to have an algorithm that finds the edge label to be added to the prefix of *C *computed up to that point. The sequence  C obtained by a depth-first visit of the tree is a sequence of edge labels whose realization results in an edgeless red-black graph [21]. Such sequence  C is called *successful c-reduction *of the red-black graph.

The rest of the paper is devoted to give a formal definition of the CPPP problem and to provide an efficient algorithm to solve that problem. Moreover we will test our algorithm on some instances that do not admit a perfect phylogeny, showing that we are able to quickly compute a persistent perfect phylogeny, hence giving a possible phylogenetic interpretation of those data.

## Results and discussion

We can now formally define the Constrained Persistent Perfect Phylogeny (CPPP) problem where the fact that a pair (*c, s*) (i.e., a character *c *and a species *s*) is constrained means that *s *and all its ancestors do not have the character *c*. The input of the problem is a binary matrix *M *and a set $F=\left\{\left(c_{i_{1}},s_{i_{1}}\right),\ldots ,\left(c_{i_{l}},s_{i_{l}}\right)\right\}$ of constraints, such as $M\left[s_{i_{j}},c_{i_{j}}\right]=0$ for each *j*. A solution for such instance is a persistent perfect phylogeny *T *for *M *such that, for each constraint $\left(c_{i_{j}},s_{i_{j}}\right)$, none of the edges from the root of *T *to the leaf labeled by $s_{i_{j}}$ is labeled $c_{i_{j}}^{+}$. This implies that no edge from the root of *T *to the leaf labeled by *s_i_j *can be labeled $c_{i_{j}}^{-}$.

The idea of the extended matrix *M_e _*applies also to the CPPP problem. In this case, if *M*[*s, c*] = 1, then *M_e_*[*s, c*^+^] = 1 and *M_e_*[*s, c^−^*] = 0, if *M*[*s, c*] = 0 and (*c, s*) is a constraint, then *M_e_*[*s, c*^+^] = *M_e_*[*s, c^−^*] = 0. Finally, if *M*[*s, c*] = 0 but (*c, s*) is not a constraint, then *M_e_*[*s, c*^+^] =? and *M_e_*[*s, c^−^*] =?. An immediate extension of the result in [21] shows that *M_e _*has a directed perfect phylogeny if and only if (*M, F*) has a constrained persistent perfect phylogeny.

Just as for the PPP problem, we first explore a graph formulation of the CPPP problem based on the equivalence of PPP to a problem of completing a matrix where each character *c *has two columns *c*^+^, *c^−^*, with *c*^+ ^(*c^−^*) equal to 1 in a species *s *in the matrix corresponds to the fact that *s *has gained (lost) the character *c*. The graph formulation derives again by representing a completion in terms of red-black graph associated to extended matrices. Notice that there exists a 1-to-1 correspondence between completing entries of the matrix and realizing characters of the red-black graph. When considering the CPPP problem, some entries of a partially completed matrix are constrained which means that some characters in the associated red-black graph cannot be realized. On the other hand, all characters in a red-black graph for the PPP problem can be realized. Thus it is quite easy to show that the main red-black graph reduction characterization stated for the PPP problem can be extended to the constrained persistent perfect phylogeny problem, by simply adding the constraint that some characters cannot be realized in a red-black graph.

Now, the red-black graph reduction turns out to be quite useful to investigate new algorithmic solutions to the PPP problem. In this paper we are able to prove that there exists a class of binary matrices that always admit a positive solution for the PPP problem, that is they admit a *persistent perfect phylogeny *that can be computed in polynomial time. For this special case we also provide a polynomial algorithm that works for the general CPPP problem. Based on this polynomial time algorithm we give a fixed-parameter (in the number of characters) algorithm for the CPPP, based on the search tree technique [24], improving the exponential time algorithm given in [21].

We observe that the CPPP problem is a special case of the General Character Compatibility problem (GCC) [19]. An instance of the GCC problem is a matrix *M_G _*having rows which are species and columns that are characters. Each entry of the matrix *M_G _*is a subset of the states that character *c *may assume in species *s*. Another part of the instance is a specification of all allowed transitions between states in a solution. A feasible solution is a perfect phylogeny where for each species *s *and for each character *c*, the state is picked from the input set *M_G_*[*s, c*]. Given an instance (*M, F*) of CPPP, we obtain a matrix *M_G _*as follows. If *M*[*s, c*] = 1, then *M_G_*[*s, c*] = {1}. If *M*[*s, c*] = 0 and (*c, s*) ∈ *F*, then *M_G_*[*s, c*] = {0}. Finally, if *M*[*s, c*] = 0 and (*c, s*) ∉ *F*, then *M_G_*[*s, c*] = {0, 2}. The only allowed transitions are from the state 0 to 1 and from 1 to 2. This case of GCC corresponds to cases 5 and 6 of Table 1 in [19], whose complexity is reported as open. Thus the results we give in the paper also apply to those cases.

**Table 1:** Running times on unconstrained simulated instances.

Species	Characters	Instances completed within 15 minutes	Min time (sec)	Max time (sec)	Average time (sec)	Standard deviation
10	5	100*/*100	0.00	0.01	0.00	0.00
10	7	100*/*100	0.00	0.25	0.01	0.03
10	10	100*/*100	0.00	1.93	0.11	0.30
10	12	94*/*100	0.00	12.95	0.84	1.93
10	15	84*/*100	0.00	43.89	5.71	9.80

20	10	100*/*100	0.00	4.72	0.08	0.47
20	15	97*/*100	0.02	18.12	1.15	2.53
20	20	93*/*100	0.13	95.03	10.44	19.14
20	25	79*/*100	1.09	253.68	41.98	60.35
20	30	63*/*100	3.84	247.03	59.06	63.81

40	20	100*/*100	0.06	89.02	2.04	8.93
40	30	98*/*100	0.99	156.16	22.03	33.17
40	40	80*/*100	7.23	598.32	128.47	154.92
40	50	45*/*100	19.14	585.42	198.81	146.39
40	60	19*/*100	50.26	577.1	319.25	183.10

60	30	99*/*100	0.64	222.79	14.36	33.21
60	45	90*/*100	8.76	590.03	123.05	148.48
60	60	51*/*100	37.63	593.06	252.34	168.92

All times are in seconds.

We recall that a main result of [21] is that finding a solution of PPP is equivalent to finding a successful c-reduction, that is a sequence of edge labels (corresponding to a depth-first visit of the tree) whose realization makes the red-black graph edgeless. For the CPPP problem a similar result holds, but we have to adapt the notion of reduction, so that there is a third case when the reduction is impossible; when for some species *s*, with (*c, s*) ∈ *F *(that is *M_e_*[*s, c*^+^] = *M_e_*[*s, c^−^*] = 0), (*c, s*) is also a red edge of *G_RB_*. Notice that, in order to obtain an algorithm to compute a persistent perfect phylogeny, it suffices to have an algorithm that finds the edge label to be added to the prefix of *C *computed up to that point.

### Solving CPPP on matrices with edgeless conflict graphs

In the following, we will exploit some properties of the red-black graph to show that a matrix *M *whose conflict graph is edgeless always admits a persistent perfect phylogeny. Moreover, we provide a polynomial time algorithm for the CPPP problem in this case.

Given *M *a binary matrix, the *partial order graph *for *M *is the partial order *P *obtained by ordering columns of *M *under the *<*relation which is defined as follows: given two character *c *and *c*', we will say that *c < c*' iff *M*[*s, c*] *≤ M*[*s, c*'] for each species *s*. Moreover, we build a graph *G *= (*V, E*), called *adjacency graph *for *M *: *V *is the set of columns of *M *and (*u, v*) is an edge of *G *if and only if *u, v *are *adjacent*, i.e. there is a species *s *that is adjacent to both *u *and *v *in the red-black graph for the extended matrix *M_e _*associated with *M*. Our algorithm for solving the CPPP problem finds a successful *c*-reduction by simply computing the maximal inactive characters in the poset *P *that can be realized in the red-black graph.

In the following we give some Lemmas that are used to show that maximal characters in the poset *P *can be realized without inducing in the red-black graph any *red-sigma graph*: this is a graph of red edges consisting of a path of length four and having two characters and three species. Such a graph represents the forbidden matrix {0, 1}, {1, 0} and {1, 1} in the completion of the extended matrix *M_e _*and thus whenever it is present in the red-black graph it means that the completion does not admit a directed perfect phylogeny [2]. In fact, by definition of red-black graph associated to a completion, a red-sigma graph corresponds to two completed characters *a*^+^, *b*^+ ^in the extended matrix such that *M_e_*[*s*_1_, *a*^+^] = 1 = *M_e_*[*s*_2_, *a*^+^] and *M_e_*[*s*_2_, *b*^+^] = 1 = *M_e_*[*s*_3_, *b*^+^], while all other entries of *M_e _*are 0 for pairs (*a*^+^, *s*_3_) and (*b*^+^, *s*_1_). The following property is easily proved by induction on the length of a path in the red-black graph connecting two maximal characters.

**Algorithm 1: **Procedure Solve-CPPP-empty-conflict

**Input **: A constrained binary matrix (*M, F*) whose associated conflict graph is edgeless.

**Output **: A realization *S_c _*of the characters of *M *resulting in a constrained persistent perfect phylogeny for (*M, F*), if such a phylogeny exists.

**1 ***S_c _← *empty sequence;

**2 ***P ← *the partial order for *M*;

**3 ***G_RB _← *the red-black graph for the extended matrix *M_e _*of *M*.

**4 while ***G_RB _is not edgeless ***do**

**5 ***C_M _← *maximal elements in *P *that are in the same connected component of *G_RB_*;

**6 ***D ← *the subset of *C_M _*consisting of the characters that can be realized;

**7 if ***D *= ∅ **then**

**8 return ***no solution*

9 else

**10 **Add to *S_c _*all characters in *D*;

**11 **Realize the characters of *D *in any order, updating *G_RB_*;

**12 **add to *D *the free characters in the graph *G_RB_*;

**Lemma 3 ***Let M be a binary matrix with an edgeless conflict graph. Assume that the extended matrix associated with M induces a connected red-black graph and let P be the partial order graph for M. Let C_M _be the set of maximal elements in P. Then C_M _consists of elements that are pairwise adjacent in the adjacency graph for M*.

The following properties can be proved by as consequences of the definition of realization of characters, and assuming that the input matrix has an edgeless conflict graph.

**Lemma 4 ***Let M be a binary matrix that has an edgeless conflict graph. Let G_RB _be the red-black graph for the extended matrix associated with M. The realization of two characters a and b that are adjacent in the adjacency graph for M adds at most two disjoint components consisting of red edges. In this case one connected component has the vertex a and the other one b*.

**Lemma 5 ***Let G_RB _be a connected red-black graph whose conflict graph is edgeless. Let C_M _be the set of maximal characters in G_RB _and let $C_{M}^{\prime }$ be the set of maximal characters in the red-black graph $G^{\prime }$ obtained after the realization of C_M_. Then: *(*1*) *the elements of C_M _are in at most two distinct connected components of $G^{\prime }$ and *(*2*) *in each of such disjoint connected component, each maximal character $c\in C_{M}^{\prime }$ is either adjacent to all species of the component or all active characters of C_M _are free*.

Notice that, the absence of conflicts does not guarantee that a solution actually exists. However, we are able to provide an efficient algorithm (Algorithm 1) for this case, which will be a cornerstone for our algorithm for the general case.

Algorithm 1 builds a successful c-reduction *S_c _*by iteratively adding to *S_c _*the maximal inactive characters or free characters of the red-black graph *G_RB_*. Notice that the successful c-reduction provides a completion of the extended matrix that admits a perfect phylogeny. The latter can be built using the classical linear time algorithm [2].

**Theorem 6 ***Let *(*M, F*) *be a binary matrix that has an edgeless conflict graph. Then Algorithm 1 computes a successful c-reduction of the red-black graph associated to the extended matrix for M, if it exists. Moreover, if F is empty then M admits a solution*.

*Proof *First observe that the correctness of Algorithm 1 is a consequence of the fact that maximal characters are realized before any character they include by the *<*-relation. Assume that *c*_1 _*< c*_2 _and let *T *be a persistent perfect phylogeny. If *c*_2 _is not persistent for *s *in *T*, then also *c*_1 _is not persistent for *s *in *T*. In fact, assume to the contrary that *c*_1 _is persistent for *s *in *T *and *c*_2 _is not persistent for *s*. This fact implies that there exists a species *s*' such that has *c*_1 _and *s*' and *s *share a common ancestor in the tree which is below edge labeled *c*^+^. Since *c*_1 _*< c*_2_, it follows that species *s*' has also character *c*_2 _and thus the edge labeled by *c^− ^*is below the edge $c_{2}^{+}$. But since *s *does not have character *c*_2 _and *c*_2 _cannot be persistent we obtain a contradiction.

We show that at each iteration of Algorithm 1 each connected component *G_RB _*has only black edges, or the connected components with red edges has no red-sigma graphs. Initially, by assumption, since no character is active, no red edge is in the connected components of the red-black graph. Then, by applying Lemma 3 and 4, the realization of the maximal characters *C_M _*of poset *P *does not induce any red sigma-graph, thus proving the invariant. Now, a successive iteration of the algorithm requires to add to *S_c _*the free characters or the maximal inactive characters of the red-black graph. By applying Lemma 5, the red-black graph has connected components without red edges or at most two components having red edges, since the active characters by statement 1 are in at most two components. For the first type of components, the invariant property is immediate since the component does not have any red edge. Consider now the second type of components. By Lemma 5, there are at most two such components, moreover, either each connected component has some maximal active character that are free or the maximal inactive are adjacent to all species of the connected component of the red-black graph. Assume that the active characters in the connected component having red-edges are free. Thus by definition, these active characters are removed from the red-black graph including all incident edges. Otherwise, the maximal active characters are all adjacent to all species and thus they are realized without adding new red edges. In both cases, the invariant property holds. Clearly, if all characters are in *S_c _*after the application of the algorithm, it is immediate that the red-black graph is edgeless since all active characters are free (no red-sigma graph is possible, indeed). Thus *S_c _*is a successful c-reduction. Observe that in case *F *is empty, all characters can be realized, and consequently, the sequence *S_c _*after the iterations of the algorithm includes all characters of the red-black graph, thus implying that a solution always exists.   □

### An algorithm for CPPP

In this section we propose an algorithm for the CPPP problem that is based on the procedure **Solve-CPPP-empty-conflict**(*M*). Our algorithm is based on the search tree technique [24], where we explore the tree of all possible c-reductions. Since in a c-reduction each signed character (*c*^+ ^or *c^−^*) can appear at most once, the search tree has at most (2*m*)! leaves. Therefore we only need to describe a polynomial-time algorithm to compute an edge of the search tree (which mainly consists of realizing a signed character).

Just as the algorithm in [21], we transform the matrix *M *of the instance (*M, F*) into an extended matrix *M_e _*which is then analyzed to find a solution. In fact, (*M, F*) has a solution if and only if there exists a successful c-reduction for *M_e _*that can be associated to a constrained perfect phylogeny. The algorithm in [21] explores all feasible permutations of the set of characters (feasible permutations means that *c^− ^*must follow *c*^+ ^and that all constraints are satisfied) of *M_e _*in order to find one that is a successful c-reduction, if such a c-reduction exists.

Clearly computing all permutation is not efficient, therefore we implicitly build a decision tree, where at each step we fix a character in a given position of the permutation. To each node *x *of the decision tree, we associate the matrix *M_e_*(*x*), obtained from *M_e _*by realizing the characters labeling the edges from the root to *x*, and its associated red-black and conflict graphs (respectively *G_RB_*(*x*), *G_c_*(*x*)). When *G_c_*(*x*) is edgeless, instead of further exploring the decision tree, we apply Algorithm 1. At the same time, if *G_RB_*(*x*) contains a red-sigma graph, then *M_e_*(*x*) does not admit a persistent perfect phylogeny. A fortiori, in that case *M_e_*(*x*) cannot admit a persistent perfect phylogeny, hence we can stop exploring that portion of the decision tree. Moreover, we can stop the search as soon as we find a solution, since we have no optimization criterion to discriminate between feasible solutions. In practice, all those criteria allow to avoid exploring a large part of the decision tree, as shown in our experimental analysis.

### Experimental analysis

We have implemented our algorithm as a C++ program and we have tested it over simulated data produced by *ms *[25]. Moreover, we have tested our program on real data coming from the International HapMap project [26]. All tests have been performed on a standard workstation.

The two different kinds of data correspond to two separate goals. The analysis on simulated data is aimed at studying the scalability of our approach for increasing numbers of species and characters. More precisely we have run our program for *n *= 10, 20, 40, 60 (recall that *n *is the number of species) and for values of *m *(the number of characters) ranging from *n/*2 to $\frac{3}{2}n$. The reason for the choice of *m *is based on some properties of all persistent phylogenies. Let *T *be a persistent perfect phylogeny consistent with a *n *× *m *matrix, and assume that the input matrix has no duplicated rows or columns. Then we can prove that *n/*2 *≤ m ≤ *2*n*.

Moreover, *ms *produces matrices that have a perfect phylogeny, but can have duplicated rows and columns. To introduce back mutations, we have randomly modified at most one state of each duplicated row. For each choice of the parameters *n *and *m *we have produced 100 random instances, on which we have run our program with a 15-minute timeout, without imposing any constraint. The results are represented in Table 1.

Then, for the first 10 of the 100 instances of each parameter choice, we have modified the input matrices, by introducing some random constraints, in order to determine if constraining the set of feasible solutions can help in finding a persistent phylogeny. For each instance of the first phase, we have produced 10 instances with 1 or 16 random constraints. For both cases we determine when at least one of the 10 constrained instances is solved more quickly than the unconstrained instance. The goal is to determine when there is a sizable (in our case 10%) probability that introducing some random constraints can help in computing a persistent phylogeny. Moreover, we determine when the median of the 10 constrained instances is solved more quickly than the unconstrained instance. In this case the goal is to determine when there is a 50% probability that some random constraints can help in computing a persistent phylogeny.

The most important result of this experiment is that for instances where our implementation requires at least a second (on average), the idea of introducing random constraints is often beneficial. This fact suggests a direction for further improvements, that is incorporating into our program some deterministic constraints, based on a cursory analysis of the conflict and of the red-black graphs. Actually, how we manage an edgeless conflict graph is as an example of this idea. Table 2 summarizes the experiment on constrained simulated instances.

Finally, the algorithm has been tested on real data coming from the International HapMap project. The data are classified by type of population. In our case, we used data from the set ASW (African ancestry in Southwest USA). Each individual is described by the two haplotypes (in our application the two haplotypes correspond to two different species, i.e. two different rows of the matrix). This experiment investigates the usefulness of the constrained persistent model to manage haplotypes data that cannot be explained by the perfect phylogeny model. In fact none of those instances admits a perfect phylogeny, but our model and implementation are able to find a reasonable interpretation to the data. The data set consists of binary matrices of dimensions 10 × 10, 26 × 15, 26 × 25, and 26 × 30. For each group we considered 10 matrices. In all cases the matrices do not admit perfect phylogeny, and the number of conflicts changes from a minimum of 4 to a maximum of 138. Increasing the size of the matrix, and therefore the number of conflicts, the percentage of matrices that admit persistent perfect phylogeny decreases. More in detail, 80% of the tested matrices of size 10 × 10 admits solution, only 20% of the tested matrices of size 26 × 15 admits solution, and none of the sets 26 × 25, and 26 × 30 admits solution. The results show that haplotype data may be related by the persistent phylogeny in case they cannot be explained by the perfect model. It would be interesting to investigate the biological soundness of the persistent perfect phylogeny in this context.

## Conclusions

The algorithms and models discussed in the paper may have interesting applications in the construction of evolutionary trees based on the analysis of binary genetic markers, where variants of the perfect phylogeny have already been considered, such as in the study of evolution based on introns [1] or progression pathways using tumor markers or in discovering significant associations between phenotypes and single-nucleotide polymorphism markers [27] and also in haplotype analysis. In this paper we have investigated the CPPP problem, which is the general problem of computing a persistent perfect phylogeny for binary matrices where some characters may be forced not to be persistent in the tree. We provide algorithmic solutions for the problem: mainly a polynomial time algorithm when the conflict graph is edgeless and a fixed-parameter algorithm. In particular we show that when no constraint is given and the conflict graph is edgeless, a solution for PPP always exists. We experimentally show that the search tree technique, combined with the use of constraints allows to obtain efficiently solutions for matrices that otherwise would require exponential time. Future research will be devoted to experimental investigation of possible improvements based on introducing a carefully crafted set of constraints to speed up the computation. The computational complexity of the CPPP problem is open and it would be interesting to solve the problem for the unconstrained case.

## Competing interests

The authors declare that they have no competing interests.

## Authors' contributions

All authors have contributed equally to the paper.

**Table 2:** Improvements of constrained simulated instances over unconstrained instances.

Species	Characters	Number of added constraints					
			1			16	
		**Fastest**		**Median**	**Fastest**		**Median**

10	5	0		0	0		0
10	7	1		0	1		1
10	10	7		5	7		7
10	12	7		5	7		6
10	15	8		3	9		8

20	10	9		4	10		10
20	15	10		9	10		10
20	20	9		1	10		10
20	25	9		7	9		9
20	30	7		2	10		9

40	20	9		7	10		10
40	30	10		7	10		10
40	40	8		1	10		9
40	50	10		0	10		10
40	60	1		0	9		6

60	30	8		7	10		10
60	45	10		8	10		10
60	60	7		6	8		7

For each choice of the number of species and of characters, we state the number of instances where at least one of the 10 random constrained instances is solved more quickly than the unconstrained instance (columns labeled Fastest). Moreover we state the number of instances where the median of the 10 random constrained instances is solved more quickly than the unconstrained instance (columns labeled Median).

## Declarations

Publication charges for this work was funded by MIUR PRIN 2010-2011 grant 2010LYA9RH.

This article has been published as part of *BMC Genomics *Volume 15 Supplement 6, 2014: Proceedings of the Twelfth Annual Research in Computational Molecular Biology (RECOMB) Satellite Workshop on Comparative Genomics. The full contents of the supplement are available online at http://www.biomedcentral.com/bmcgenomics/supplements/15/S6.
